# Closure of Orocutaneous Fistula Using Submandibular Gland as a Pedicled Flap

**DOI:** 10.1155/2019/3438626

**Published:** 2019-02-27

**Authors:** Erol Cansiz, Emine Deniz Gozen, Murat Yener

**Affiliations:** ^1^Istanbul University, Faculty of Dentistry, Department of Oral and Maxillofacial Surgery, Turkey; ^2^Istanbul Cerrahpasa University, Medical Faculty, Department of Otorhinolaryngology, Turkey

## Abstract

Orocutaneous fistulas in the maxillofacial region may be due to tumor resection, osteoradionecrosis, or trauma, and these defects limit the function of the patients and effect aesthetic and also psychological condition. Articulation and nutrition are also affected by these fistulas. Local flaps can be used for the reconstruction of small- and medium-sized defects with ease. Submandibular gland with its rich blood supply from the facial artery is a practical and useful choice for the reconstruction of mandibular region defects.

## 1. Introduction

Orocutaneous (or orofacial) fistula is a pathological pathway between the oral cavity and the cutaneous surface of the face. The most common causes of the orocutaneous fistulas are malignancy, inflammation, and trauma, and they may lead to functional, aesthetic, and psychological problems due to the irrepressible leakage of saliva from the oral cavity to the face [1].

Literature search on this topic, by using “orocutaneous, fistula, closure” keywords, does not reveal convincing results about treatment options or incidence. There are few reports about the management of orocutaneous fistula. Balakrishnan et al. successfully used pedicled expanded deltopectoral flap to repair fistula in a patient with previous neck dissection and radiotherapy [2]. Another method is tissue plug technique in which the dermal component of a pedicle flap is advanced through the fistula, and with external traction, the tissue obliterates the tract [3]. Tian et al. used a vacuum-assisted closure system for the management of orocutaneous fistula and were able to treat most of their patients [4]. There are other techniques for the closure of orocutaneous fistulas. Local rotational flaps and advancement flaps, transpositional flaps, autogenous, alloplastic, or allogenic grafting techniques may be used for the reconstruction [5]. Each technique has pros and cons, and it is important to choose the right technique in the right situation.

As most of the orocutaneous fistula closes by conservative methods like antibiotic therapy, decrease of salivary secretions, packing, and nasogastric tube feeding, the orocutaneous fistulas are not regarded as a major complication. However, in the situation of a persistent fistula, great discomfort may occur both for the patient and the operator [6]. In this technical note, the closure of a resistant orocutaneous fistula by using submandibular gland as a pedicled flap is presented.

## 2. Case Report

**Figure 1:**
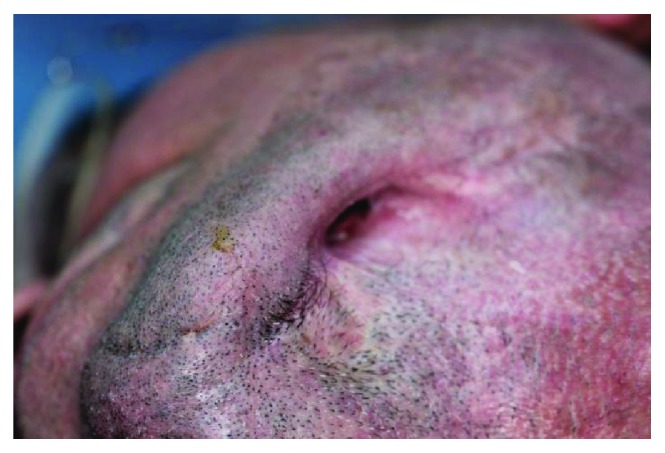
Pre-op view of the orocutaneous mandibular fistula from the bottom.

**Figure 2:**
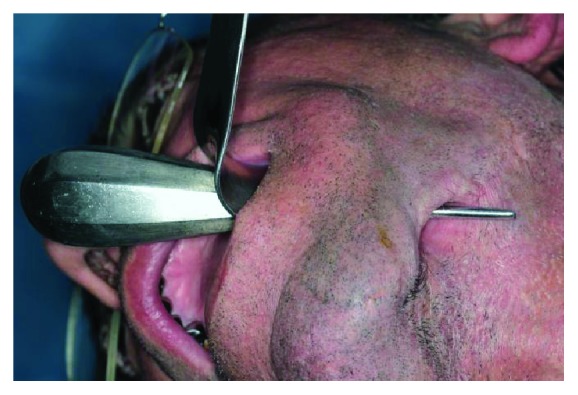
Figure shows the relation of defect with oral cavity.

**Figure 3:**
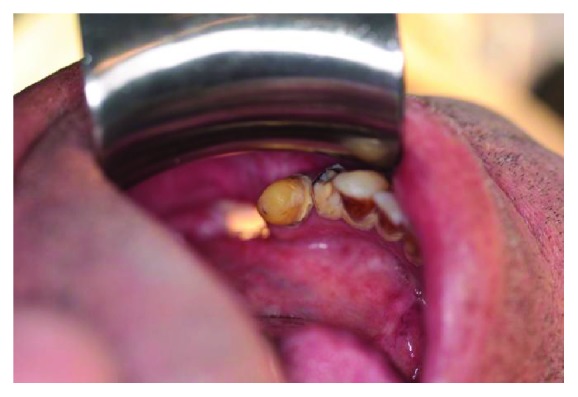
Intraoral view of the fistula; the light from outside can be seen through the defect hollow.

A 56-year-old male patient, whose plasmacytoma was enucleated 3 years ago from the medial side of the left mandible, was referred to our clinic due to his complaints of persistent orocutaneous fistula (Figures 1–3). Past medical history revealed that the patient was operated three times to close the orocutaneous fistula by local flaps; however, none of these operations were successful. CT images of the patient demonstrated that the medial side of the left mandible was missing and there was a 3 × 2 cm diameter defect located between the left side of the mouth floor and the basis of the left mandible neighboring the left submandibular gland. The main reason of the failed attempts to close the fistula was considered to be the ineffective management of dead space surrounding the fistula. Consequently, it was decided to use the submandibular gland as a pedicled flap to fill the defect and support the oral and the cutaneous flaps.

**Figure 4:**
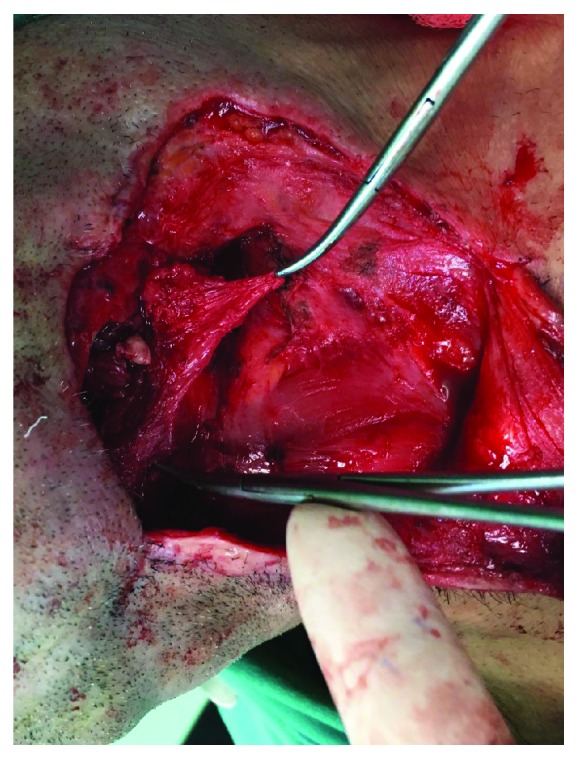
The rotation of the submandibular pedicled flap to the defected site.

**Figure 5:**
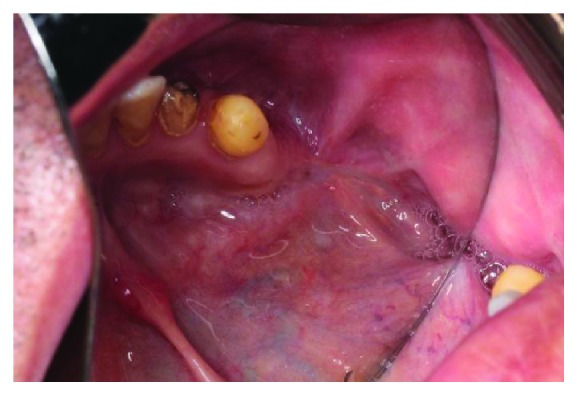
Post-op 6 months; closure of the fistula could be seen from the intraoral view.

**Figure 6:**
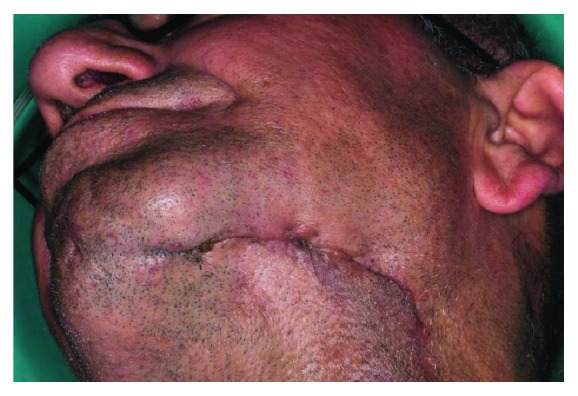
Extraoral operation site in 6 months.

Under general anesthesia, the fistula was excised initially and the oral and the cutaneous healthy soft tissues were prepared. At the extraoral site, the incision was extended to the posterior and anterior directions, following the previous incision lines. In the subplatysmal plane, the superficial layer of the neck fascia was dissected to reach the base of the mandible. After the dissection of the fascia, the submandibular gland and the base of the mandible were exposed, the soft tissues surrounding the submandibular gland were dissected, and the gland was mobilized by protecting the arteriovenous supply and the duct. At the oral site, the margins of the wound were released by blunt dissection and were closed by mattress sutures via 5/0 polypropylene. Following the mobilization of the gland, a soft tissue tunnel was prepared between the submandibular space and the defect area and the submandibular gland was rotated by passing the gland through the soft tissue tunnel by preserving the pedicle (Figure 4). The rotated gland was sutured to the recipient site with 3/0 reabsorbable polyglaction sutures for the stabilization. At the cutaneous site, the flap was closed layer by layer by using 3/0 resorbable polyglaction for the facia and the subcutaneous layers and 3/0 polypropylene sutures for the skin. After the surgery, pressure bandage was applied for the edema control externally and the patient was ordered soft diet for a week. The postoperative healing was uneventful, and at the sixth month follow-up visit, ideal closure of the fistula was observed (Figures 5 and 6).

## 3. Discussion

An orofacial or orocutaneous fistula is a pathological communication between the cutaneous surface of the face and the oral cavity. These defects can cause aesthetic and functional problems due to the continuous leakage of saliva. The common causes are malignancy, osteoradionecrosis (ORN), residual lesions of the cyst and tumors of the jaws, inflammation, and trauma [5].

The reconstruction of the defects in the maxillofacial region should be based on the principles of function and aesthetic. For this aim, free flaps, pedicled flaps, local regional flaps, and rotation flaps can be used. Local flaps have some advantages like providing the same skin texture and color, and also morbidity is less because there is no other donor site [7].

Submandibular gland flap which has its own blood supply from facial artery was first introduced by Mozolewski et al. [8] in the reconstruction following partial laryngectomy. Yang et al. [9] used the submandibular gland flap successfully for the reconstruction of the defects in the infratemporal region after tumor resection. In addition, Zhang et al. reported the usage of submandibular gland flap for the reconstruction of oral cavity [10].

In general, if the donor site is far from the recipient site, the success of the local flap can be hazardous. The pedicle of the flap should be handled and placed gently without any tension. The pedicled submandibular gland flap has some advantages like being near the orofacial zones so it can be rotated to a desirable place without tension. With this type of flap, spaces (like defects or fistulas) can be filled with minimal morbidity and no loss of function of the gland.

In comparison to allografts, the submandibular flap has no risk of tissue rejection, no potential transmission of disease from the donor of the allograft, or no prolonged healing response.

It also has some pros when comparing with free flaps. The main disadvantage of a free flap depends on the technical difficulties related to the microsurgery [11]. In free flaps, if the revascularization is unsuccessful flap, failure is inevitable. The presence of two operation sites and increased operation time is the other cons of the free flaps. Rotation of the pedicled submandibular gland flap is accomplished from the same incision, and there is no need to wait for the revascularization or microsurgery.

On the contrary, as the pedicle of the submandibular gland flap is relatively short, the flap is suitable in the submandibular region. Careful and rigorous surgery must be performed in order to avoid gland obstruction which results in functional loss. Ranula formation and atrophy of a gland are other possible complications of this flap. The pedicled submandibular gland flap is a good choice in defects near the mandibular region, and it is also a good alternative when other rotational flaps failed.
